# The Influence of the Evolutionary Past on the Mind: An Analysis of the Preference for Landscapes in the Human Species

**DOI:** 10.3389/fpsyg.2018.02485

**Published:** 2018-12-07

**Authors:** Joelson M. B. Moura, Washington S. Ferreira Júnior, Taline C. Silva, Ulysses P. Albuquerque

**Affiliations:** ^1^Laboratório de Ecologia e Evolução de Sistemas Socioecológicos (LEA), Departamento de Botânica, Universidade Federal de Pernambuco, Recife, Brazil; ^2^Programa de Pós-Graduação em Etnobiologia e Conservação da Natureza, Departamento de Biologia, Universidade Federal Rural de Pernambuco, Recife, Brazil; ^3^Colegiado de Biologia, Universidade de Pernambuco, Petrolina, Brazil; ^4^Departamento de Biologia, Universidade Estadual de Alagoas, Santana do Ipanema, Brazil

**Keywords:** evolutionary psychology, social-ecological systems, emotional response, human evolution, savanna hypothesis

## Abstract

According to some evolutionary psychologists, landscapes preferences in the human species are influenced by their evolutionary past. Because the Pleistocene savanna is the least inhospitable landscape, it was the most suitable environment for survival and influenced the evolution of hominids in such a way that even today the human being has a universal preference for these environments. However, there is controversy regarding this statement, because in some studies it was evidenced that people prefer images of landscapes that are similar to those of the environment where they live. In this sense, we want to test whether there is indeed a preference for images of the savanna landscape and how the current environmental context may influence this preference. We performed a study in three environmental contexts with different landscapes in order to be able to observe the influence of the familiar landscape on landscape preference, of which two rural communities — one presenting a landscape similar to the deciduous seasonal forest and another presenting a savanna-like landscape — that totaled 132 participants and one urban community with 189 participants. The stimulus consisted of 12 images representing the six major terrestrial biomes and two images of urban landscapes. The variables analyzed were the emotional responses and the preference of the participants in relation to the images of landscapes. We analyzed the data using the Kruskal–Wallis test. The obtained result did not corroborate the idea of universal preference for images of savanna landscape. The image of Rainforest landscape was the preferred one among all the three environmental contexts studied. In this way, the preference for landscape may have been shaped at different periods of human evolutionary history, and not just during the period when hominids lived on the savannah. As much as selective pressures of the Pleistocene savanna have shaped the human mind during the evolutionary history, other factors and different types of environments may have influenced human preferences for landscapes. Thus, evolutionary psychologists who analyze human preferences for images of landscapes, guided by the idea of the past influencing the present, must be cautious before generalizing their results, especially if other variables such as the cultural ones are not controlled.

## Introduction

Some studies suggest that the selective pressures imposed on early hominids in Africa during the Pleistocene, specifically in the savanna environments, were so decisive for the evolutionary history of the human species that, to date, there seems to be a universal preference for this type of landscape (Orians and Heerwagen, 1992; Falk and Balling, 2010; Klasios, 2016; Townsend and Barton, 2018). Some authors often struggle to test this assertion (see Sommer, 1997; Han, 2007; Falk and Balling, 2010; Hartmann and Apaolaza-Ibáñez, 2010, 2013). However, there is controversy regarding this argument because in some studies, it was evidenced that people prefer images of landscapes similar to those of the environment in which they live (Balling and Falk, 1982; Lyons, 1983; Van den Berg et al., 1998).

The idea of the past influencing preferences and, consequently, human behavior at present has been the basis of several investigations in the area of evolutionary psychology. For example, several studies attempt to understand sexual preferences in partner selection (Schwarz and Hassebrauck, 2012; Conroy-Beam and Buss, 2018), preference for objects (Carbon, 2010; Altman et al., 2016), and food preferences (Hasford et al., 2018), among others.

A set of studies has found evidence in support of the preference for images of savanna landscapes (Orians and Heerwagen, 1992; Sommer, 1997; Falk and Balling, 2010; Townsend and Barton, 2018). In other cases, such a preference was not observed. In these studies, it was evidenced, for example, that people living in Spain, when analyzing photographs of advertisements in natural and urban environments, tend to prefer and to express positive emotional responses to images of landscapes of exuberant green forests, which are typical of that country (see Hartmann and Apaolaza-Ibáñez, 2010, 2013).

Other countries where landscape preference was evaluated include Nigeria – in which savanna landscape images were preferred (Falk and Balling, 2010); Zimbabwe, South Africa, Estonia, Italy, and Switzerland - in which images of trees with broad crowns and branched trunks were preferred (Sommer, 1997); Australia – in which the typical landscapes of the country were preferred (Herzog et al., 2000); and the United States – in which images of trees whose shapes are characteristic of the savanna, with broad crowns and branched trunks, were preferred (Summit and Sommer, 1999). However, in the study of Han (2007) performed with American university students, among the six great terrestrial biomes, the savanna and desert images were the least preferred and tundra and coniferous forest images were the most preferred.

In addition, in some of these studies, methodological biases can be identified, for example: (i) the use of images that did not encompass the six large terrestrial biomes — i.e., desert, tundra, savanna, coniferous forest, deciduous forest and rainforest; (ii) the use of ambivalent scales to measure preference forced people to choose between something only positive or negative; (iii) the use of landscape images with the presence of clean water — water alone evokes positive feelings such as pleasure and tranquility (Ulrich, 1983); and (iv) the use of landscapes with different shades of blue in the sky, which is considered a universally preferred color (Saad and Gill, 2000). Thus, the controversial results of the abovementioned studies, when added to the methodological biases, do not allow for understanding if, in fact, the selective pressures that occurred in the past have developed a universal preference for images of savanna landscapes in humans.

Thus, we used certain essential ideas of evolutionary psychology — such as the *evolved psychological mechanisms* — to understand the particular mechanisms that precede human behavior, solving the mentioned methodological limitations. To achieve this goal, we conducted two empirical experiments that sought to analyze the preference and emotional responses of people living in different environmental contexts to images of landscapes located in terrestrial biomes.

## Conceptual Framework

### Human Preferences for Images of Landscapes

Orians (1980) elaborated a hypothesis that would unleash a series of empirical studies (e.g., Balling and Falk, 1982; Lyons, 1983; Orians and Heerwagen, 1992; Sommer, 1997; Summit and Sommer, 1999; Falk and Balling, 2010; Hartmann and Apaolaza-Ibáñez, 2010, 2013) named the *savanna hypothesis*. According to this hypothesis, there is a universal affinity in the human species to prefer open landscapes similar to the savanna. This preference occurs because the savanna possesses a combination of environmental conditions that facilitated the survival and reproductive success of early hominids in the Pleistocene (Appleton, 1975; Orians and Heerwagen, 1992; Sommer, 1997; Summit and Sommer, 1999). For example, although the savanna is an open landscape, it contains shrubs and sparse trees, offering a combination of perspective and refuge which may have solved the specific problem of the identification of predator approach, provided better mobility, and provided potential hiding places (Appleton, 1975; Kaplan, 2001; Townsend and Barton, 2018).

The preference for savanna is considered to be an evolved domain-specific psychological mechanism which processes information from the environment and evolves by solving particular adaptive problems that early hominids encountered under ancestral conditions (Buss, 1995; Tooby and Cosmides, 2015). In this sense, adaptive problems — such as confronting a predator — were very different and specific, and therefore required specific solutions to each problem, stimulating the mind to work as computer software that individualized specific mental modules — or specific domains — in order to solve problems (Buss, 1995; Tooby and Cosmides, 2015; Klasios, 2016).

This idea is based on empirical evidence of characteristics of human behavior that exhibit a universal pattern, such as: facial recognition of relatives; the fear of snakes, spiders, darkness, height and strangers; child care; sexual attraction to partners who show kindness and intelligence; and the detection of cheaters in everyday situations, among others (see Buss, 1995; Tooby and Cosmides, 2015; Townsend and Barton, 2018). These modules were inherited by humans, and they exist because they solved specific adaptive problems of survival or reproduction of early hominids and are only activated by certain specific environmental information (see Buss, 1995).

Thus, based on the savanna hypothesis (Orians, 1980) and the findings of some empirical studies on the preference for images of savanna landscapes (Orians and Heerwagen, 1992; Summit and Sommer, 1999; Falk and Balling, 2010), we tested the following hypothesis:

H1: The preference for images of savanna landscapes is universal in the human species. Thus, people living in different environmental contexts demonstrate a significantly greater preference for images of savanna landscapes than for any other type of landscape.

In studies that tested the preference for images of landscape, some obtained results that do not corroborate the savanna hypothesis. In some cases, people preferred images of landscapes similar to the environmental context in which they live (Balling and Falk, 1982; Lyons, 1983; Van den Berg et al., 1998; Hartmann and Apaolaza-Ibáñez, 2010). According to some authors, an explanation for these results would be that, although there is a universal preference for savanna configurations, preferences are modified due to the particular ontogenetic development of each individual, which may lead to a preference for images of familiar landscapes (Balling and Falk, 1982; Lyons, 1983). These findings suggest that even if there are universal preferences, sociocultural values and the environmental context people live in may have a part in the influence on how a person will prefer and respond emotionally to the environment (Korpela et al., 2002). According to Tuan (1980), cultural pluralism fosters different cosmovisions, leading people to respond to environmental stimuli differently from one another. These responses are influenced by aspects of the landscape that are appealing to people (Ulrich, 1983) such as familiarity, for example. In this sense, we alternatively tested the following hypothesis:

H2: The human preference for images of landscapes depends on the environmental context in which people live. Thus, people living in different environmental contexts demonstrate a higher preference for images of landscapes that are similar to the environment in which they live than for other types of landscapes.

### Emotional Responses in the Human Species

According to the assumptions of evolutionary psychology, the human mind is understood as an integrated structure of several evolved psychological mechanisms that regulate behavior and were selected because they solved several adaptive problems (Al-Shawaf et al., 2015; Tooby and Cosmides, 2015). These mechanisms — for example, preferences and emotions — can interact with each other by working in coordination when confronted with the most diverse adaptive problems (Al-Shawaf et al., 2015; Tooby and Cosmides, 2015) to improve fitness (Klasios, 2016). In this sense, an important role of emotions in the evolutionary past, especially in foraging activities and predator avoidance, was to help reduce energy expenditure by avoiding unnecessary actions (see Eisend, 2018).

Adaptive problems, such as confronting a predator, for example, require a subset of instructions that regulate and guide the most appropriate behavior to deal with the situation. This structured functioning of the set of mechanisms is interpreted in evolutionary psychology as an emotional state, and the specific feeling that this state will manifest is the signal that will activate a cascade of appropriate mechanisms to solve the adaptive problem — such as preferring a specific landscape, for example (Al-Shawaf et al., 2015; Tooby and Cosmides, 2015).

In this sense, emotions can regulate various psychological mechanisms (Cosmides and Tooby, 2000). For example, a person contemplating images of a savanna landscape may feel happy and secure, and these feelings may work in combination with the universal preference for savanna, interacting with perceptual mechanisms. Because we believe that by preferring an image of a landscape, a person also elicits emotional responses that are congruent with such preference, we find it logical to test the following hypotheses:

H3: The images of savanna landscapes provoke positive emotional responses in people. Thus, more positive feelings will be evoked for images of savanna landscapes than for any other kind of landscape.

To observe the influence of familiar landscapes, we alternatively tested the following hypothesis:

H4: The images of landscapes similar to those in which people live activate positive emotional responses. Thus, more positive feelings will be evoked for landscapes that are similar to the environmental context that people live in than for other types of landscapes.

## Materials and Methods

### Participants and Experimental Design

**FIGURE 1:**
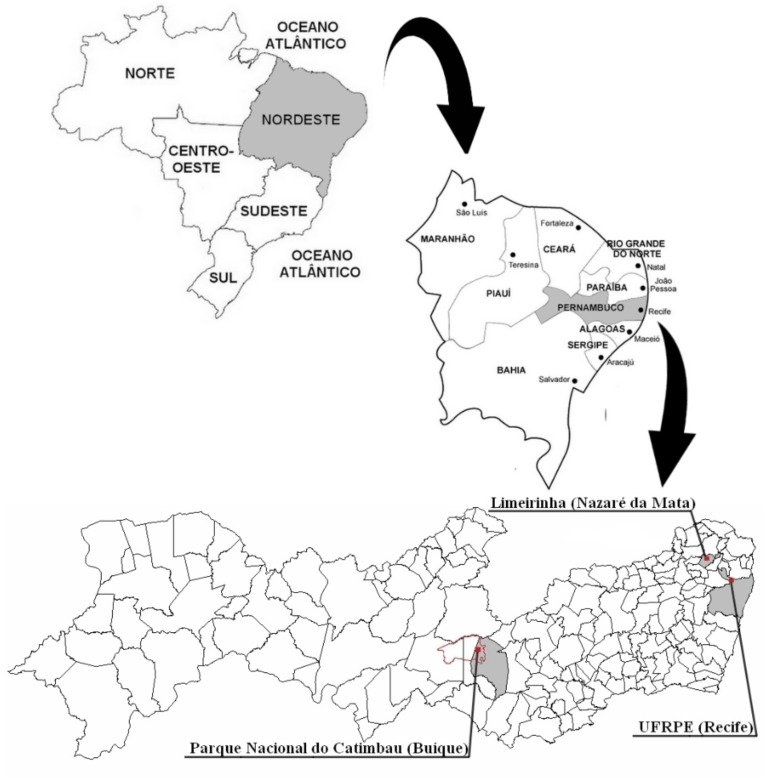
Location of the three municipalities of the state of Pernambuco that were included in the study. The red line demarcates the entire length of the Parque Nacional do Catimbau, one of the sites investigated, although our study covered only the region of the park that belongs to the municipality of Buíque.

We performed an empirical study in three environmental contexts with different landscapes — Atlantic forest, caatinga and urban landscape — all located in the state of Pernambuco, Northeast region of Brazil (Figure 1). The Research Ethics Committee involving human beings of the Universidade Federal de Pernambuco approved this study (decision number 1.727.669). All participants read and signed the Free and Informed Consent Form, which explains the procedures and purpose of the research.

The purpose of investigating three distinct environmental contexts was precisely to observe if the experimental factor of the familiar biome would lead people to prefer landscapes that are similar to the environment where they live. Thus, the environmental contexts analyzed were equivalent to some landscapes used in this study. For example, people living in the context of the Atlantic Forest — specifically the context of the Atlantic Forest chosen in our study, which is a semideciduous forest — are familiar with landscapes that are similar to deciduous seasonal forests; people living in the urban context are familiar with urban landscapes; people living in the Caatinga are familiar with savanna-like landscapes. In this sense, the Caatinga exhibits at least 13 different phytophysiognomies, including savanna (see Silva et al., 2017), in which the landscape near the communities investigated in our study resembles those of the savanna photographs that we used, with bushy vegetation and sparse trees.

We selected the maximum number of people who agreed to participate in the survey and were over 18 years old and literate.

**FIGURE 2:**
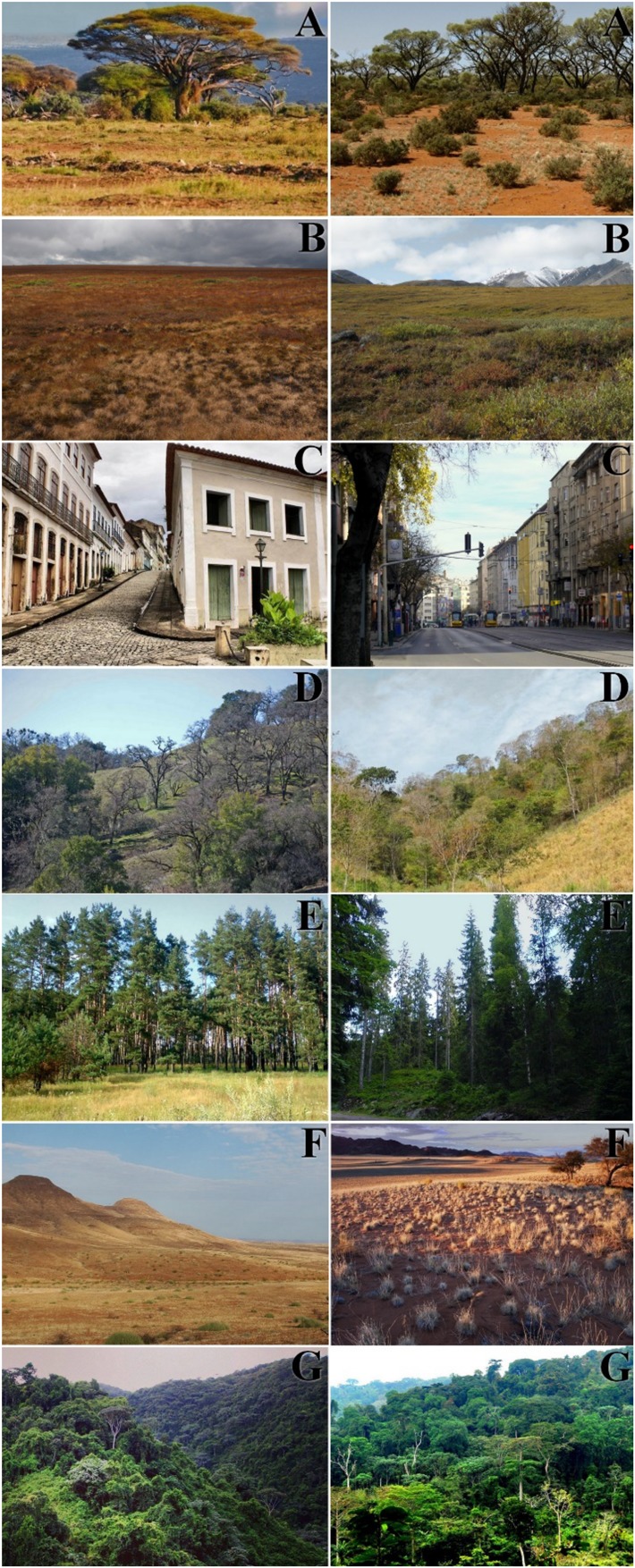
Images of the 14 landscapes used in the experiment. **(A)** Savanna; **(B)** tundra; **(C)** urban; **(D)** deciduous seasonal forest; **(E)** coniferous forest; **(F)** desert; **(G)** rainforest. Source: https://pixabay.com/en/photos/?q=savannah&image_type=&cat=&min_height=&min_width=&order=popular&pagi=2%22 https://pixabay.com/en/photos/?hp=&image_type=&cat=&min_width=&min_height=&q=tundra&order=popular https://pixabay.com/en/photos/?hp=&image_type=&cat=&min_width=&min_height=&q=city&order=popular https://pixabay.com/en/photos/?q=deciduous+forest&image_type=&cat=&min_height=&min_width=&order=popular&pagi=2 https://pixabay.com/en/photos/?q=coniferous+forest&image_type=&cat=&min_height=&min_width=&order=popular&pagi=2 https://pixabay.com/en/photos/?hp=&image_type=&cat=&min_width=&min_height=&q=desert&order=popular https://pixabay.com/en/photos/?q=tropical+forest&image_type=&cat=&min_height=&min_width=&order=popular&pagi=2.

We adapted the methods used in the studies by Hartmann and Apaolaza-Ibáñez (2010, 2013) that analyzed whether people preferred and elicited more positive emotional responses to images of savanna, urban scenarios or dense vegetation scenarios. We used pairs of photographs printed on matte paper (23 × 19 cm) with 14 images. We defined the order that photographs were presented for preference evaluation based on a sortition executed by the software BioEstat 5.3, and we presented the images in the same order to all participants, for each of the following landscapes: an urban city scenario and biomes of the savanna, tundra, desert, rainforest, conifer forest and deciduous seasonal forest (Figure 2). The purpose of using pairs of photographs was to make people’s assessment of landscapes more consistent. The photographs represented all the great terrestrial biomes according to the classification of Odum (1989). The choice of this classification was to make our study more standardized and replicable, since there are several classifications for terrestrial biomes, and the criteria of these classifications are often not clear. Some authors, for example, classify the terrestrial world into 14 biomes (Olson et al., 2001).

Additionally, none of the images exhibited animals, water or different shades of blue sky. The images were edited using the software *PhotoFiltre Studio* X; the purpose of editing the images was to make the blue color of the sky less distinct between the photographs. Selection of the images, photos with good photographic quality, minimal distortion, horizontal layout, and representation of the six large terrestrial biomes and urban landscapes, was based on the criteria of Han (2007).

The Brazilian tropical dry forest (Caatinga) sample consisted of 50 participants who were born and live inside the Parque Nacional do Catimbau (PNC), which is georeferenced by the coordinates 8°30′12^′′^ and 37°22′14^′′^, located 11 km from the center of the municipality of Buíque. The ages of participants ranged from 18 to 72 years, of which 62% were female and 38% were male. Approximately 800 inhabitants live within the PNC, according to data provided by the local health office in December 2016. The main economic activities of local residents are agriculture and goat breeding. The PNC has a territorial extension of approximately 62.000 hectares and a specific ecosystem of Brazil known as caatinga, which is a mosaic of seasonally dry forests and shrub vegetation (Pennington et al., 2009).

The Brazilian Atlantic forest sample consisted of 82 participants who were born and live in the rural community of Limeirinha, which is georeferenced by the coordinates 7° 44′ 28^′′^ and 35° 10′ 50^′′^, located 6.5 km from the center of the municipality of Nazaré da Mata. The ages of participants ranged from 18 to 84 years, with 66% women and 34% men. The community of Limeirinha has approximately 269 inhabitants distributed in 80 houses, according to data provided by the local health office in October 2015. The main economic activity of the residents is family agriculture, especially of cassava, beans and corn. In addition to agriculture, there is also the rural work of the cutting of sugar cane. The community is surrounded by a semideciduous Atlantic forest landscape — a transitional forest — known as Mata da Alcaparra, which is accessed by residents to collect firewood.

The urban sample was represented by undergraduate and graduate students from the Universidade Federal Rural de Pernambuco (UFRPE) — located in the city of Recife, the capital of the state of Pernambuco. The students from the university who volunteered to participate in the survey but came from rural communities or lived in rural places were excluded from the sample. Thus, the sample was formed by 189 participants who were born and live in urban contexts. The ages of the participants ranged from 18 to 51 years, with 54% female and 46% male. Of the students participating in the research, 49% were undergraduates of the Biological Sciences course, 8% were undergraduates of the Fisheries Engineering course, 6% were postgraduate students and the remaining 37% were distributed among the courses of Administration, Veterinary Medicine, History, and Sociology, among others.

Participants were recruited in the urban site through electronic forms via the *Survey Monkey* website and in rural communities through direct contact with each volunteer. After the recruitment of participants from the urban context, we directed each individual to a separate UFRPE classroom to perform the experiment. The experiment with participants from Limeirinha and CNP was performed in their own homes individually and without the presence of family or friends, to avoid possible biases in the results. For all participants, the stimuli were presented by the researcher, who showed the images one by one for appreciation and evaluation by the participants.

### Measurements

The emotional responses evoked by the images were classified into six categories of basic emotions — pleasure, enthusiasm, happiness, freedom, security and interest — as presented by Hartmann and Apaolaza-Ibáñez (2010), on a semantic differential scale ranging from 1 to 5 — for example, 1 indicates sadness and 5 indicates happiness (see Appendix A). These emotional dimensions are effectively measurable and are evoked by exposure to images of the environments (see Hartmann and Apaolaza-Ibáñez, 2010). Each participant evaluated only one image, randomly selected among 14 landscape images — randomization was applied using the Bioestat 5.0 program — and were then asked to evaluate their emotional responses to the presented landscape based on the semantic differential scale. The evaluation of only one image was made to avoid comparisons, aiming the analysis at spontaneous emotions. The photograph was exhibited by the researcher himself.

After apprehending emotional responses, we measured the participants’ preference for images of the landscape. For this, we adapted the method of Hartmann and Apaolaza-Ibáñez (2010). The adaptation was made to avoid ambivalence, that is, to avoid people feeling forced to respond as only liking or disliking the landscapes. Thus, we used a *Likert-type* scale which varied from 10 to 1, anchored by the following classification of appreciation: I liked it a lot, I liked it, Neutral, I did not like it and I did not like it at all (see Appendix A). Each participant was exposed to all 14 landscape images (Figure 2 and asked to mark, based on the *Likert-type* scale, how much they liked each landscape presented.

### Data Analysis

To analyze the preferences and emotional responses of the participants toward images of landscapes, we utilized the Kruskal–Wallis test using the statistical program “R” (R Core Team, 2016). We separated and organized the scores that the participants assigned for preference and emotional response toward each of the 14 images of landscapes using Microsoft Excel software. Finally, score sets were compared through the test to evaluate whether there was a significant difference in preferences and emotional responses. After the analysis, we performed a Dunn *post hoc* test to verify where the difference was in the values.

Emotional responses were analyzed at two levels: (i) positive emotional responses made by the person were observed by adding the values of the six feelings that the participant attributed to each landscape, and scores ranged from 6 to 30 — the higher the value of the sums of scores, the greater the positive emotional response to the landscape —, and (ii) the emotional responses of the three surveyed sites were separated by category of feeling and analyzed.

## Results

### Preference for Images of Landscapes

Rainforest was the preferred landscape in all three environmental contexts studied. This result did not corroborate hypotheses H1 and H2 because this landscape is also not familiar to participants in any of the contexts studied. The results of the descriptive analysis of preference for images of landscape in all environmental contexts are shown in Table 1.

**Table 1:** Median differences (Kruskal–Wallis) relative to preference of the images of landscapes and the descriptive analysis of the preference of the participants from the three environmental contexts studied.

Analyzed environment	*N*	*H*	Landscape type	Median	Mean	Standard deviation
Urban city	189	379.56^∗^	Coniferous forest	8	8.16	1.60
			Deciduous forest	7	7.01	1.95
			Desert	7	6.91	2.17
			Savanna	8	7.41	1.98
			Rainforest	10	8.94	1.45
			Tundra	8	7.29	2.24
			Urban	7	6.65	2.01
Caatinga	50	128.64^∗^	Coniferous forest	10	8.82	1.96
			Deciduous forest	8	7.26	2.70
			Desert	7	6.54	2.63
			Savanna	8	7.55	2.24
			Rainforest	10	9.37	1.33
			Tundra	7	6.43	3.01
			Urban	8	7.44	2.46
Atlantic forest	82	173.87^∗^	Coniferous forest	9	8.44	2.04
			Deciduous forest	7	6.95	2.36
			Desert	6	5.94	2.76
			Savanna	8	7.46	2.28
			Rainforest	10	8.85	1.87
			Tundra	7	6.29	2.87
			Urban	8	7.32	2.57

*^∗^*p* < 0.001*.

**FIGURE 3:**
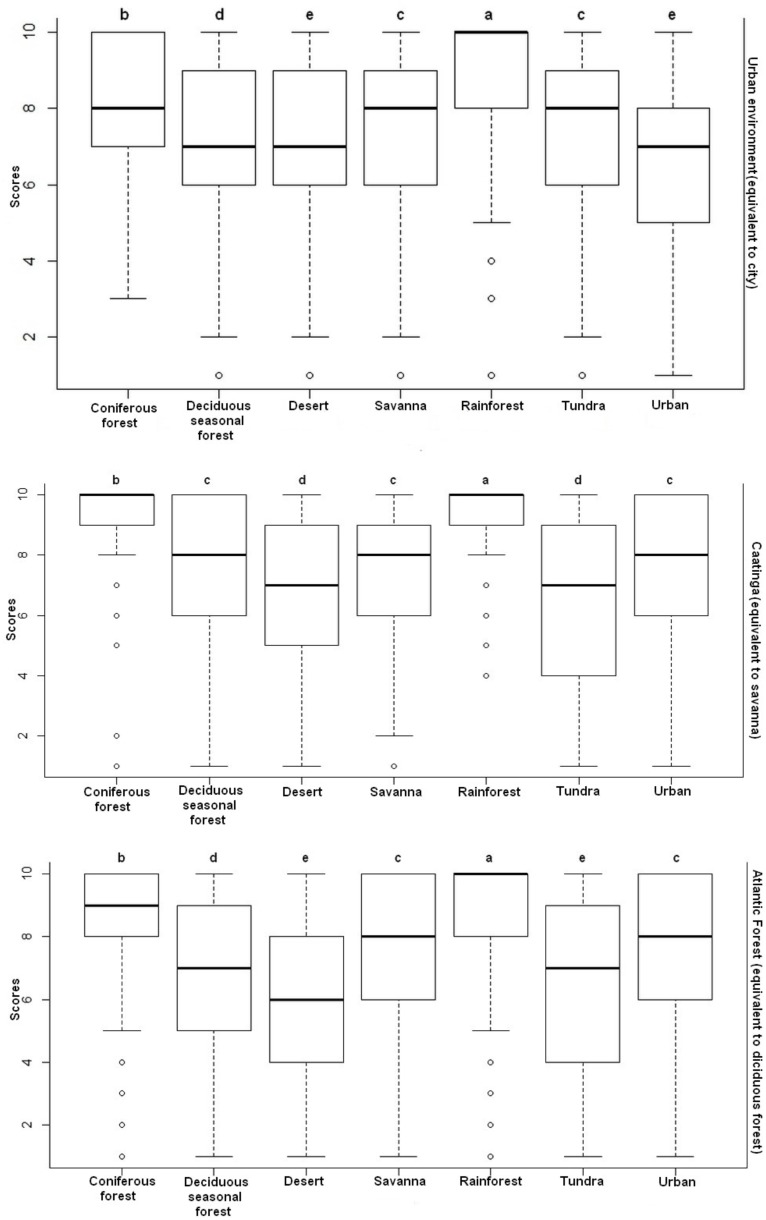
Distribution of the scores for the preferences toward images of landscapes attributed by the participants of the three environmental contexts investigated. Equal letters for *p* > 0.05 represent non-significant differences; different letters for *p* < 0.05 represent significant differences.

The comparison of the values showed a significant difference in the preference toward images of landscapes of the people living in the three distinct environmental contexts: in the Caatinga (*H* = 128.64; *p* < 0.001), in the Atlantic forest (*H* = 173.87; *p* < 0.001) and in the urban environment (*H* = 379.56; *p* < 0.001). The Dunn *post hoc* test showed that rainforest was the most preferred landscape image (*p* < 0.05) in relation to any other type of landscape image (Figure 3).

### Emotional Responses to the Images of Landscapes

The images of landscapes that most elicited positive emotional responses were the rainforest and conifer forest. These results do not corroborate hypotheses H3 and H4 because these landscapes are also not familiar to the participants in any of the contexts studied. The results of the descriptive analysis of emotional responses for images of landscapes in all environmental contexts are shown in Table 2.

**Table 2:** Median differences (Kruskal–Wallis) in emotional responses toward images of landscapes and the descriptive analysis of emotional responses of participants from the three environmental contexts studied.

Analyzed environment	*N*	*H*	Landscape type	Median	Mean	Standard deviation
Urban city	189	41.61^∗^	Coniferous forest	24	23.31	5.29
			Deciduous forest	20	20.53	4.98
			Desert	20	18.48	4.48
			Savanna	21	20.51	4.32
			Rainforest	25	24.78	3.98
			Tundra	20	19.32	5.23
			Urban	17	17.06	4.78
Caatinga	50	22.76^∗^	Coniferous forest	24	24.50	3.77
			Deciduous forest	22	18.85	8.64
			Desert	22	22.60	4.98
			Savanna	24	21.71	7.84
			Rainforest	28	28.11	1.96
			Tundra	16	15.75	5.17
			Urban	16	15.50	6.83
Atlantic forest	82	10.92	Coniferous forest	25	22.90	6.80
			Deciduous forest	19	21.81	6.35
			Desert	15.5	15.70	6.41
			Savanna	17.5	18.20	8.82
			Rainforest	23	21.09	6.71
			Tundra	16	16.93	8.21
			Urban	13	15.71	7.52

*In this analysis, we added the scores attributed to the six feelings; therefore, the values ranged from 6 to 30. The higher the value of the median, the more positive the emotional response. ^∗^*p* < 0.001*.

**FIGURE 4:**
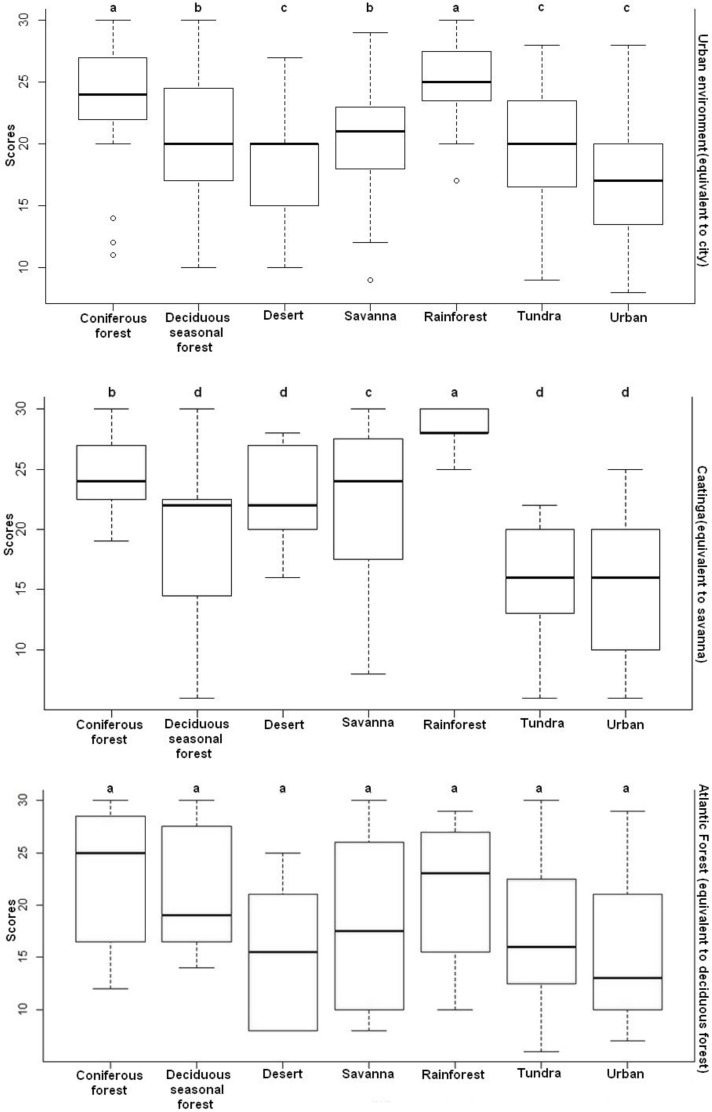
Distribution of the scores for the emotional responses toward images of landscapes attributed by the participants of the three environmental contexts investigated. The values of the *y*-axis represent the sum of the scores of the six basic emotions: pleasure, happiness, enthusiasm, interest, safety, and freedom. The higher the value, the more positive the emotional response. Equal letters for *p* > 0.05 represent non-significant differences; different letters for *p* < 0.05 represent significant differences.

The comparison of the values showed a significant difference in the emotional responses toward images of landscapes of the people living in the caatinga (*H* = 22.76, *p* < 0.001) and in the urban environment (*H* = 41.61; *p* < 0.001). However, for the Atlantic forest environment, the Kruskal–Wallis test was non-significant (*H* = 10.92; *p* > 0.05). The Dunn *post hoc* test showed that the rainforest and the conifer forest were the landscapes that made people in the urban environment feel more positive emotions (*p* < 0.05). In the context of the caatinga, the rainforest image elicited more positive emotional responses (*p* < 0.05) (Figure 4).

#### Emotional Responses to the Images of Landscapes by Category of Feeling

The image of landscapes that most elicited positive emotional responses, separated by category of feeling, were the rainforest and conifer forest. These results did not corroborate hypotheses H3 and H4 because these landscapes are not familiar to the participants in any of the contexts studied.

The comparison of the values showed a significant difference for the feelings of “pleasure” (*H* = 61.35; *p* < 0.001), “interest” (*H* = 27.48; *p* < 0.001), “happiness” (*H* = 53.43; *p* < 0.001), and “enthusiasm” (*H* = 31.92; *p* < 0.001) in relation to the three environmental contexts studied. With this, we verified by the Dunn *post hoc* test that the rainforest and the conifer forest were the landscapes that elicited these feelings in people (*p* < 0.05).

In the case of the feelings of “safety” (*H* = 17.28; *p* < 0.001) and “freedom” (*H* = 67.16; *p* < 0.001), the comparison of the values also showed a significant difference. The Dunn *post hoc* test showed that the rainforest was the landscape that elicited these feelings in people (*p* < 0.05).

Because our sample was proportionally different between the male and female participants, especially in the Atlantic Forest and Caatinga contexts, we performed a complementary analysis to observe the effect of gender. Our results showed that densely green and closed forests are the preferred landscapes among people, regardless of gender (see Appendix B). However, in the Caatinga and Mata Atlântica environments, there was not a prominent landscape in relation to emotional response except for the participants from the urban environment, who provided more positive emotional responses for dense and green forests, regardless of gender (see Appendix B).

## Discussion

This study consisted of a careful comparison that rejected the evolutionary psychology hypothesis on a universal preference for savanna landscape due to the evolutionary past of humans. The savanna was not the most preferred landscape, but rather the rainforest, a similar result found by Hartmann and Apaolaza-Ibáñez (2010, 2013). Our findings conclusively show that there is no evolutionary preference for this type of landscape. New studies should investigate the reasons for the preference for rainforests, since our experimental design does not allow us to infer about it.

The savanna hypothesis and the alternative hypotheses were not corroborated. Therefore, our results support the evidence that there is no preference for images of savanna landscapes (Han, 2007; Hartmann and Apaolaza-Ibáñez, 2010, 2013). The fact that early hominids originated and evolved in the savanna environment did not generate universal preferences in humans for images of open landscapes similar to the savanna, as has been suggested by some authors (Appleton, 1975; Orians, 1980; Townsend and Barton, 2018). In addition, the environment in which people have developed and live in does not influence their preferences for images of landscapes, as some studies suggest (Balling and Falk, 1982; Lyons, 1983; Van den Berg et al., 1998).

One of the explanations for this result may be the influence of culture. Although people live in different environmental contexts, they all live in Brazil, and the various media information about the Amazon rainforest, which is extremely dense and closed, may influence people’s preferences. However, this has not been tested. Thus, without isolating cultural variables, we cannot safely conclude that the preference for dense and closed landscapes in the human species is universal, as some studies suggest (Kaplan and Kaplan, 1989; Korpela et al., 2001; Cackowski and Nasar, 2003). Another explanation may be the influence of perceived naturalness^1^, as abundant vegetation is an important component in the regulation of visual preferences due to their degree of naturalness (Purcell and Lamb, 1998). Thus, to state clearly that there are universal factors that direct people to prefer forests with dense and closed vegetation, it is necessary to execute another study that attempts to isolate the influence of the media and the environmental context on preferences.

If at some point in our evolutionary history the human mind worked as it is predicted by the savanna hypothesis, it is most likely that social, cultural, and media influences have shaped human preferences of images of savanna environments. In this sense, culture can exert an influence on how people perceive the environment (Tuan, 1980; Shepard, 2004; Goldstein, 2010).

Our findings also suggest the possibility that early hominids evolved in closed rainforests during the Pleistocene (Andrews, 1989; Roberts et al., 2016). There is evidence, such as the discovery of an early Pleistocene *Homo* fossil in a rainforest in Southeast Asia (Roberts et al., 2016) and the knowledge of certain foraging activities of prehistoric humans (Barker et al., 2007), which suggest an adaptation of early hominids to the rainforest environment. However, the idea of human origin in rainforests must be considered with caution for several reasons: (i) the evidence that bipedalism arose in the savanna (Rodrigo, 2014); (ii) the relative scarcity of archeological research in these scenarios; and (iii) the fact that many archeologists and anthropologists understand rainforests as barriers to the expansion of hominids, which makes these scholars prioritize the role of open savanna environments in the evolutionary history of the human species (see Foley et al., 2016; Roberts et al., 2016).

In addition, humans may have developed other psychological mechanisms during their evolutionary history in a period prior to the establishment in the savanna or in a later period of emigration. This may have generated preferences for images of extremely green landscapes (see Hartmann and Apaolaza-Ibáñez, 2010, 2013), for example.

Our findings do not invalidate the argument that our mind is a product of the past (see Tooby and Cosmides, 2015). Although we have evolved as a species in the African Pleistocene savanna (Rodrigo, 2014), humans may not have inherited a universal preference for images of this environment, which is considered a specific evolved psychological mechanism, but may have developed general psychological mechanisms in response to the various selective pressures offered in the Pleistocene (see Buller and Hardcastle, 2000; Bolhuis et al., 2011; Young et al., 2012). In this sense, the human mind would be equipped with only a few general cognitive procedures used to learn everything it came to know about the world — such as in the case of language acquisition and mathematical ability — and these minimal procedures are called *general modules* or *general domains* of the human mind (Buller and Hardcastle, 2000).

Recent evidence suggests, for example, that humans respond adaptively to a survival situation depending on the type of threat, regardless of whether the threat belonged to an ancestral environmental context — in this case, the savanna — or to a contemporary environmental context (Young et al., 2012; Yang et al., 2014). Therefore, what may have been selected for in humans was not the preference for a specific landscape but the ability to develop survival strategies regardless of the type of environment, suggesting the existence of mechanisms of more general domains (Yang et al., 2014). However, we have not tested this theory in this research.

Regarding emotional responses, the images of landscapes with closed forests caused people in the urban and caatinga context to elicit positive feelings. However, the fact that there are no extreme emotional responses created by images of landscapes in the context of the Atlantic forest but there is a preference for images of the rainforest landscape, suggests that the preference for images of landscape is not always linked to the emotional responses of people to these landscapes. That is, psychological mechanisms, such as preferences and emotions, do not always interact as some authors propose (Al-Shawaf et al., 2015; Tooby and Cosmides, 2015). This can also be observed in the urban environment group, where the emotional responses to images of conifer forest landscape predominate, although it was not prominently considered as the preferred landscape. This evidence may mean that some assumptions of evolutionary psychology must be revisited because emotional responses to images of landscapes and their relations to preferences may vary between human groups living in distinct environmental contexts, suggesting that culture may be modeling universal behaviors (Goldstein, 2010).

In addition, by analyzing the emotional responses separated by category of feeling, the images of landscapes of closed forests also made people elicit positive feelings such as enthusiasm, happiness, interest and pleasure. In the case of feelings of freedom, and especially of security, feelings were more elicited for images of rainforest landscapes. This may indicate that the savanna does not necessarily reflect a landscape that elicits greater security to early hominids, as evolutionary psychology assumes (Orians and Heerwagen, 1992).

The findings of the present study suggest that people’s emotional response to and preference for a habitat or landscape is not solely influenced by psychological mechanisms shaped in a specific environment. However, these results should be taken with some caution, once the data were collected in a single country, which limits the generalization to other countries.

Although selective pressures of the ancestral savanna have shaped the human mind during evolutionary history, other factors, and not only those from biological evolution, may exert a selective role that promotes faster evolution than that at the genetic level (see Laland and Brown, 2006), currently influencing the psychological mechanisms of human beings. Thus, evolutionary psychologists who analyze human preferences, guided by the idea of the past influencing the present, must have some caution before generalizing their results, especially if cultural variables, for example, are not controlled.

### Limitations and Future Research

The main limitation of this study possibly implicates the lack of control on other factors that may influence people’s emotional responses and preferences in relation to the displayed landscape images. For example, according to Ode et al. (2009), the use of photographs is a very useful tool to analyze preferences for landscapes, but there can often be an influence of intrinsic variables on each image. Some aesthetic factors of the images may influence human perception, such as perceived naturalness (see Ode et al., 2009), the complexity of the landscape — the amount of information contained in the landscape (see Han, 2007) — and the perceived disturbance, among others (to better understand these factors, see Lee and Son, 2017). In addition, the images are devoid of organoleptic aspects inherent to landscapes — such as smell, humidity, temperature, among others — and this can be a limiting factor.

In this sense, evolutionary aesthetics is an important scientific field that begins with the evolutionary perspective to understand the influence of aesthetic aspects in how we react to various phenomena (Rusch and Voland, 2013). Although evolutionary aesthetics attempts to explain our aesthetic preferences — particularly human physical attractiveness (see Rusch and Voland, 2013) — based on our evolutionary past, we have guided our study of theories and concepts of evolutionary psychology to examine whether the savanna landscape would be the preferred landscape and would act independently of the aesthetic elements that form it.

The self-selection that we applied to recruit participants also has limitations that can generate some degree of bias. However, this form of recruitment is widely performed in research that uses controlled experiments because volunteers are required to be willing to participate in the research and undergo the experiment, which requires time, even though there is no benefit from their participation (see, for example, studies by Nairne et al., 2007; Young et al., 2012).

Moreover, because the preferred landscape in our study was the rainforest and the participants lived in the same country which has a strong mediatic appeal to the conservation of the Amazon rainforest^2^, we suggest that future research analyzes the influence of the media on human perception, more specifically in relation to the preference for images of landscape. This can have important practical implications because cultural information may be directing the manner in which a person responds to certain environmental stimuli. In addition, we suggest that participants should be asked if they are already familiar with a particular landscape image as an approach to control the influence of the media in preference.

## Author Contributions

UA, TS, and WJ contributed conception and design of the study. JM organized the database, performed the statistical analysis and wrote the first draft of the manuscript. All authors contributed to manuscript revision, read and approved the submitted version.

## Conflict of Interest Statement

The authors declare that the research was conducted in the absence of any commercial or financial relationships that could be construed as a potential conflict of interest.

## Footnotes

1 Naturalness can be understood as how close a landscape is to its natural state, from the perception of a person (Ode et al., 2009).

2 https://amazoniasocioambiental.com.br/2016/08/ongs-da-amazonia/
