# Supplementary material for: The Influence of the Evolutionary Past on the Mind: An Analysis of the Preference for Landscapes in the Human Species
Source: Front Psychol. 2018 Dec 7;9:2485. doi: 10.3389/fpsyg.2018.02485 (PMC6292944; doi:10.3389/fpsyg.2018.02485)
Supplement: Supplementary file 1 [file Table_1.docx]

**The Influence of the Evolutionary Past on the Mind: An Analysis of the Preference for Landscapes in the Human Species**

Joelson M.B. Moura, Washington S. Ferreira Júnior, Taline C. Silva, Ulysses P. Albuquerque.

**Appendix A: Form used to measure emotional response and preference toward landscapes**

**Emotional Responses**

Pleasure Unpleasure

5 4 3 2 1

Enthusiasm Discouragement

5 4 3 2 1

Happiness Sadness

5 4 3 2 1

Freedom Imprisonment

5 4 3 2 1

Safety Unsafety

5 4 3 2 1

Interest Disinterest

5 4 3 2 1

**Preferences**

Neutral

I did not like it

I liked it a lot

I liked it

I did not like it at all

10

9

8

7

6

5

4

3

2

1
