# Supplementary material for: The Influence of the Evolutionary Past on the Mind: An Analysis of the Preference for Landscapes in the Human Species
Source: Front Psychol. 2018 Dec 7;9:2485. doi: 10.3389/fpsyg.2018.02485 (PMC6292944; doi:10.3389/fpsyg.2018.02485)
Supplement: Supplementary file 2 [file Table_2.docx]

**The Influence of the Evolutionary Past on the Mind: An Analysis of the Preference for Landscapes in the Human Species**

Joelson M.B. Moura, Washington S. Ferreira Júnior, Taline C. Silva, Ulysses P. Albuquerque.

**Appendix B: Complementary analyzes for preferences and emotional responses to observe the effect of gender.**

**Caatinga context**: emotional response to the landscapes

*Men*

The Kruskal-Wallis test was non-significant (H=9.95; *p* >0.05).

*Women*

The Kruskal-Wallis test was non-significant (H=12.44; *p* >0.05).

**Caatinga context:** preference to the landscape

*Men*

The Kruskal-Wallis test was significant (H=55.69; *p* <0.05). The Dunn post-hoc test showed that the rainforest was the most preferred landscape (*p* <0.05).

*Women*

The Kruskal-Wallis test was significant (H=76.80; *p* <0.05). The Dunn post-hoc test showed that the rainforest and the conifer forest was the most preferred landscape (p<0.05).

**Atlantic forest context:** emotional response to the landscapes

*Men*

The Kruskal-Wallis test was non-significant (H=8.51; *p* >0.05).

*Women*

The Kruskal-Wallis test was non-significant (H=7.91; *p* >0.05).

**Atlantic forest context:** preference to the landscapes

*Men*

The Kruskal-Wallis test was significant (H=63.11; *p* <0.05). The Dunn post-hoc test showed that the rainforest was the most preferred landscape (*p* <0.05).

*Women*

The Kruskal-Wallis test was significant (H=113.87; *p* <0.05). The Dunn post-hoc test showed that the rainforest and the conifer forest was the most preferred landscape (*p* <0.05).

**Urban context:** emotional response to the landscapes

*Men*

The Kruskal-Wallis test was significant (H=21.64; *p* <0.05). The Dunn post-hoc test showed that the rainforest was the one that made men feel more positive emotions (p <0.05).

*Women*

The Kruskal-Wallis test was significant (H=27.73; p <0.05). The Dunn post-hoc test showed that the rainforest and the conifer forest was the ones that made women feel more positive emotions (p <0.05).

**Urban context:** preference to the landscapes

*Men*

The Kruskal-Wallis test was significant (H=184.44; *p* <0.05). The Dunn post-hoc test showed that the rainforest was the most preferred landscape (*p* <0.05).

*Women*

The Kruskal-Wallis test was significant (H=258.07; p <0.05). The Dunn post-hoc test showed that the rainforest was the most preferred landscape (*p* <0.05).
